# Usefulness of the Coronary Artery Calcium Score in Predicting Subsequent Coronary Interventions—A Ten-Year Single-Center Perspective

**DOI:** 10.3390/ijerph16122132

**Published:** 2019-06-16

**Authors:** Agnieszka Mlynarska, Rafal Mlynarski, Maciej Sosnowski

**Affiliations:** 1 Department of Gerontology and Geriatric Nursing, School of Health Sciences, Medical University of Silesia, 40-635 Katowice, Poland; 2 Department of Electrocardiology, Upper Silesian Medical Centre, 40-635 Katowice, Poland; joker@mp.pl; 3 Department of Electrocardiology and Heart Failure, School of Health Sciences, Medical University of Silesia, 40-635 Katowice, Poland; 4 Unit of Noninvasive Cardiovascular Diagnostics, Upper Silesian Medical Centre, 40-635 Katowice, Poland; maciej.sosnowski@gmail.com; 5 3rd Division of Cardiology, Medical University of Silesia, 40-635 Katowice, Poland

**Keywords:** CACS, calcium score, coronary artery disease, atherosclerosis, follow-up

## Abstract

There is no consensus as to whether the Coronary Artery Calcium Score (CACS) results can affect the therapeutic approach that is selected for coronary artery disease. The aim of this study was to follow patients’ management over a period of ten years after application of the CACS. *Methods*: The research was conducted as a prospective, single-center, long-distance study. In 174 asymptomatic patients (78M; aged 58.9 ± 7.86), a CACS examination using 64-slice computed tomography was performed between 2008 and 2009. The patients were divided into three subgroups according to the CACS results using Agatston Units (AU)—G1: CACS = 0 AU (52 pts); G2: CACS = 1–399 AU (64 pts) and G3: CACS ≥ 400 AU (58 pts). During the ten years of follow-up, the classical cardiovascular risk factors, drugs, diseases, and information about the therapeutic approach that was used (PCI—Percutaneous Coronary Intervention; CABG—Coronary Artery Bypass Graft) were also analyzed. *Results*: The average time until a percutaneous intervention (PCI) was 825.2 ± 1111.7 and for CABG, it was 529.0 ± 833.6. PCI was performed in 5.8% (G1), 4.7% (G2) and 32.6% (G3) of the cases, respectively; *p* = 0.0000. CABG was performed in 0% (G1), 1.6% (G2) and 18.9% (G3) of the cases, respectively; 0.0035 Yates. The area under the curve in PCI was 0.783 (95% CI: 0.714–0.841); in CABG, it was 0.825 (95% CI: 0.760–0.878) and the average for both groups was 0.838 (95% CI: 0.774–0.889). *Conclusions*: The coronary artery calcium score can potentially help to predict the best therapeutic approach for coronary artery disease in a ten-year perspective.

## 1. Introduction

A coronary artery calcium score (CACS) examination is a non-invasive examination of the coronary arteries in which the amount of calcium in the coronary arteries is determined using cardiac computed tomography [1,2]. Agatston et al. developed this method of calculation and the Agatston score is currently the standard for measurements during a semi-automatic analysis [1,3,4]. With the exception of patients with renal failure, who may also have medial calcification, coronary calcium is exclusively the result of coronary atherosclerosis. The amount of calcium in the arteries roughly correlates with extent of any atherosclerotic plaque that is present in the coronary arteries [5]. According to the European Guidelines on cardiovascular disease prevention in clinical practice (2016 version), the CACS can be considered for cardiovascular risk assessment in asymptomatic adults who are at a moderate risk [6]. The coronary artery calcium score, which is calculated in cardiac computed tomography, can support a cardiovascular risk evaluation, and therefore, it can support clinical decisions. Interestingly, Japanese researchers confirmed that an elevated CACS that is determined using coronary computed tomography angiography is an independent predictor of mid- to long-term cardiovascular mortality and morbidity in patients that are suspected of having coronary artery disease (CAD) [7]. There is still no consensus as to how the CACS results can affect the therapeutic approach to coronary artery disease. The aim of the study was to follow patients’ management over a period of ten years after use of the CACS.

## 2. Methods

The presented research was designed as a single-center observational study. We included 174 consecutive patients (average age 58.9 ± 7.9) in the long-distance study including:

- 103 (57.5%) women aged 50 to 65 years
- 71 (42.5%) men aged 40 to 65 years

All of the subjects that were included were asymptomatic, and a suspicion of coronary artery disease was the basis for being qualified for a CACS examination. Patients who were pregnant or lactating, claustrophobic, and anyone with significant heart rhythm disorders were excluded from the research. Patients who had previously had a myocardial infarction, a coronary artery bypass graft (CABG), percutaneous coronary intervention (PCI), or renal impairment were also excluded as well as patients with other diagnosed serious diseases that could interfere with the quality-of-life results. The coronary artery calcium score examination was performed using 64-slice computed tomography.

The patients were divided into three subgroups according to their CACS results in Agatston Units (AU):

G1: CACS = 0 AU (52 patients);
G2: CACS = 1–399 AU (64 patients);
G3: CACS ≥ 400 AU (58 patients).

The Medical University of Silesia Ethics Committee approved the study protocol (KNW/0022/KB1/133/09). The study protocol complied with the version of the Helsinki Convention that was current at the time the study was designed. All of the procedures that were performed in studies involving human participants were done in accordance with the ethical standards of the institutional and/or national research committee and with the 1964 Helsinki declaration and its later amendments or comparable ethical standards. Informed consent was obtained from all of the participants that were included in the study.

### 2.1. CACS Methods

Computed tomography was performed using an Aquilion 64 scanner (Toshiba Medical Systems, Ōtawara, Tochigi Prefecture, Japan) between 2008 and 2009. The scanning with prospective ECG-gating was performed during a breath-hold using 64-slices with a collimated slice thickness of 3 mm. A breath-hold typically lasted seven–eight seconds. The final reconstructions of the data were performed on Vitrea 2 workstations (Vital Images, Minnetonka, MN, USA; software versions 3.9.0.0 and 5.1). Calcification was calculated using the Agatston scale and “2DVScore with Color” semiautomatic presets by two experts who were trained in multi-slice computed tomography of the heart (performing more than 300 calcium score examinations annually). The coronary arteries were selected manually by experienced researchers. 

### 2.2. Follow-Up

During the ten years of follow-up, the classical cardiovascular risk factors, drugs, diseases, and information about the therapeutic approach (PCI—Percutaneous Coronary Intervention; CABG—Coronary Artery Bypass Graft) were also analyzed.

### 2.3. Statistical Analysis

In order to check the normality of the data distribution, the Shapiro–Wilk test was used. Comparisons of the two groups were performed using the Student’s *t*-test when there was a normal distribution of a variable in the groups being analyzed or using the U Mann–Whitney test for any distributions that were not normal. The distributions were quantified using a χ^2^ test. A ROC (Receiver Operating Characteristic) curve analysis was used to evaluate the diagnostic performance of the CACS. The area under the curve was calculated to reflect and to compare the predictive value of the CACS in order to discriminate patients that had a coronary intervention. Event-free from PCI and/or CABG survival between the patients in the groups according the CACS results is presented using the Kaplan–Meier method. The results were considered to be significant at a *p*-value of <0.05. All of the presented analyses were performed using MedCalc (MedCalc Software, Ostend, Belgium).

## 3. Results

The characteristics of the patients that were included are presented in Table 1. Weight and BMI was statistically higher in the patients in the CABG-treated group. The most prevalent risk factor was arterial hypertension, whose presence was statistically higher in the CABG group. It is worth mentioning that diabetes was the most common risk factor in the PCI group but not in the CABG group.

The average follow-up period was 2291.4 ± 1360.7 days. The average time until percutaneous intervention (PCI) was 825.2 ± 1111.7 days, and until CABG, it was 529.0 ± 833.6. PCI was performed statistically (*p* = 0.0000) more often in the G3 group, which had a CACS ≥400 AU. The percentage distribution was 5.8% in G1, 4.7% in G2 and 32.6% in G3, respectively. CABG was also performed more frequently in the G3 group—the percentage distribution was 0% in G1, 1.6% in G2, and 18.9% in G3, respectively; 0.0035 with the Yates’ correction.

**Figure 1:**
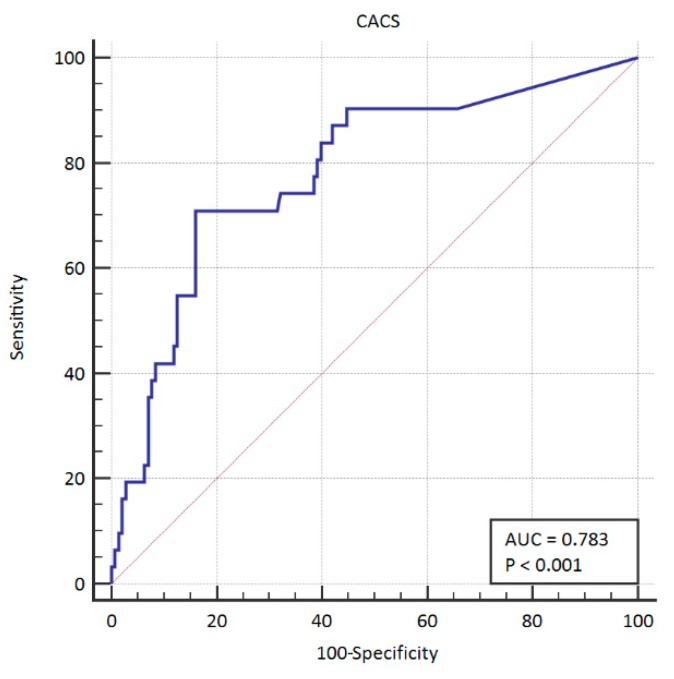
ROC curve for CACS in PCI. ROC: receiver operating characteristics; PCI: percutaneous coronary intervention.

**Figure 2:**
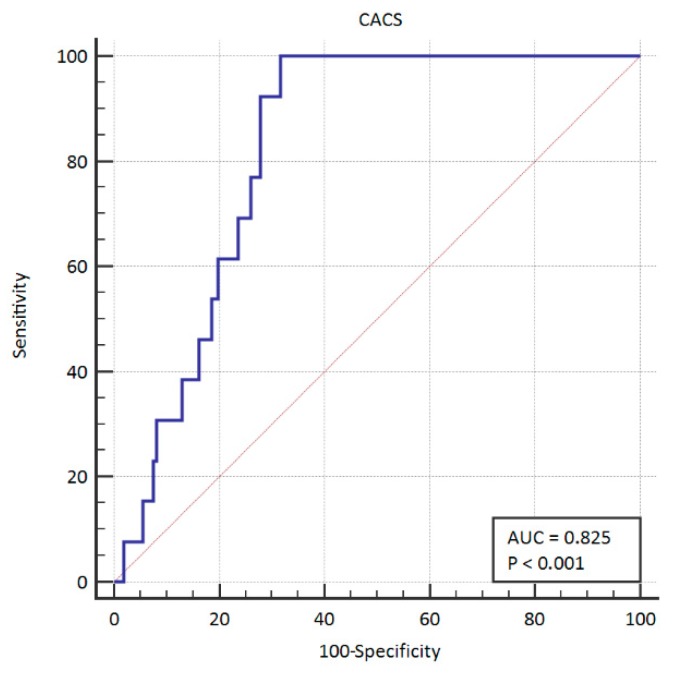
ROC curve for CACS in CABG. ROC: receiver operating characteristics; CABG: coronary artery bypass surgery.

**Figure 3:**
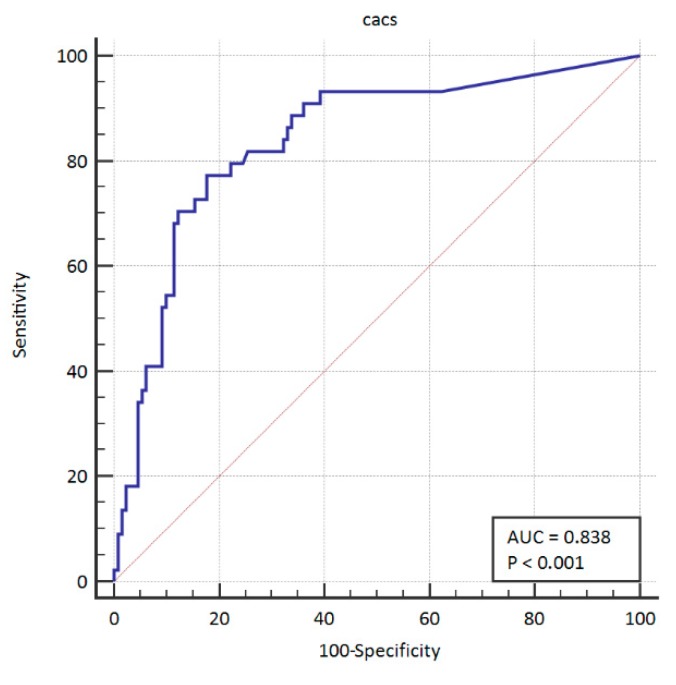
ROC curve for CACS in PCI + CABG together. ROC: receiver operating characteristic; PCI: percutaneous coronary intervention; CABG: coronary artery bypass surgery.

The area under the curve for PCI was 0.783 (95% CI: 0.714–0.841). This ROC curve is presented graphically in Figure 1. The area under the curve for CABG was 0.825 (95% CI:0.760–0.878). The ROC curve is presented graphically in Figure 2. We also summarized both groups in order to create a group of patients in which coronary artery disease was treated invasively—in this case. the area under the curve was 0.838 (95% CI: 0.774–0.889). The ROC curve in this case is presented graphically in Figure 3.

**Figure 4:**
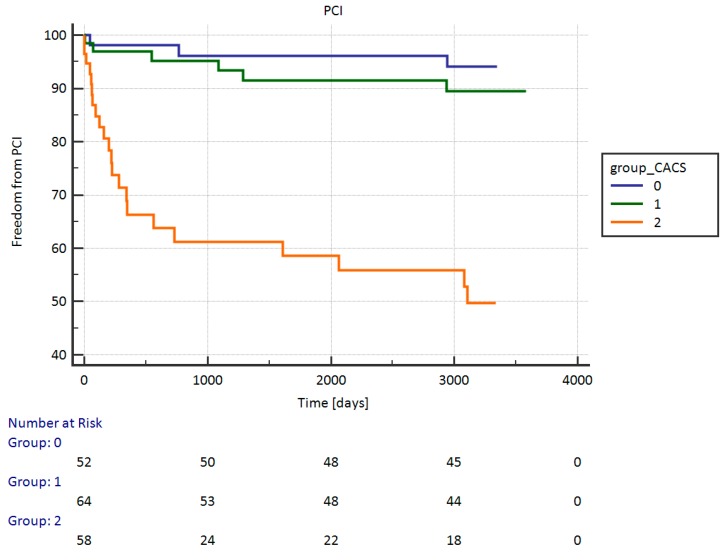
Event-free from PCI survival between patients with groups according to the CACS results. PCI: percutaneous coronary intervention.

**Figure 5:**
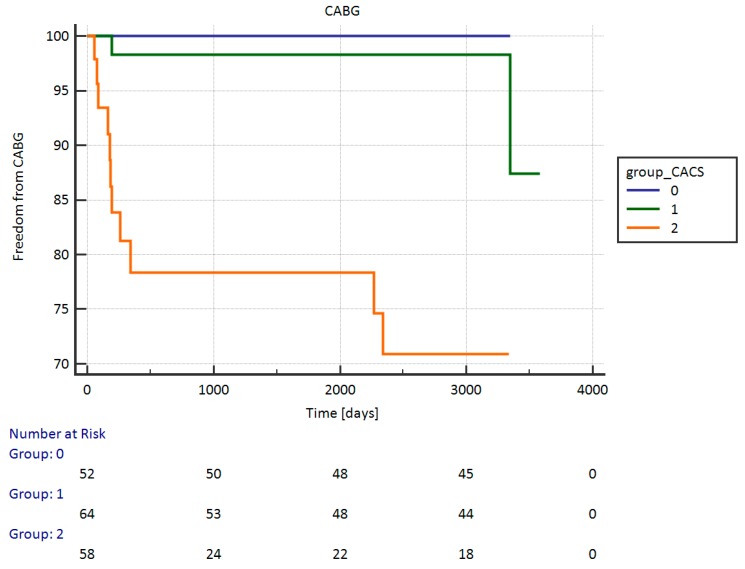
Event-free from CABG survival between patients with groups according to the CACS results. CABG: coronary artery bypass surgery.

The Kaplan-Meier survival analysis showed a statistical (*p* < 0.0001) difference in event-free from PCI survival between the groups of patients according to the CACS results—results are graphically presented in Figure 4. Interestingly, we also found a statistically significant *p* < 0.0001 frequent event‑free CABG survival in patients who had a lower coronary artery calcium score—see Figure 5. There were also statistically significant differences in the event‑free PCI and CABG survival among patients with other results of the coronary artery calcium score; *p* < 0.0001. The Kaplan-Meier survival analysis also showed a statistical (*p* = 0.0006) difference in the event-free from PCI survival between women and men. No differences were found between the event-free from CABG and both (PCI and CABG). In the logistic regression, the coronary artery calcium score results were an independent predictor of PCI (OR: 1.0009, 95% CI: 1.0003–1.0015; *p* = 0.0015), but not of CABG. 

## 4. Discussion

Within the last few years, the role of the coronary artery calcium score has changed, and this has been reflected in guidelines [8,9]. However, the question of precisely how the results of a CACS study affect the further treatment of those patients remains. One of the biggest studies on the CACS was the MESA analysis [10]. Asymptomatic patients (*n* = 3923) who had CAC scores of 0 to 10 were examined. It was found that CACS = 0 AU was observed in 3415 individuals, whereas 508 had a CACS between one and ten. During a medium-term follow-up (4.1 years), there were 16 strong cardiovascular events and 28 general CHD events in individuals with an absent or minimal CAC. In our research, percutaneous intervention was performed in only 5.8% of the patients and CABG was not performed in any of the patients.

Based on the long-term analysis, a few risk calculators were created. One example of these is the MESA—Multi-Ethnic Study of Atherosclerosis (MESA)—the authors created an algorithm for using the calcium score results, which was based on 6814 patients that were analysed during a ten-year follow-up [11]. After the calcium score results was added to the MESA risk score, the authors achieved significant improvements in risk prediction, which was confirmed by external validations in both the HNR (Heinz Nixdorf Recall Study) and the DHS (multi-ethnic, population-based, cohort study of Dallas County adults). The authors concluded that an accurate estimate of the ten-year CHD risk could be obtained using the traditional risk factors and the calcium score. Similarly, the Hartaigh team created prognostication tools that used the coronary artery calcium scoring to predict mortality [12]. An analysis was created based on 9715 individuals for which a CACS was created. The authors confirmed that their easy-to-use nomogram effectively predicted the 5-, 10- and 15-year survival for asymptomatic adults who were undergoing screening for cardiac risk factors. These are just two examples of developing methods in which the addition of the CACS result supported the predictive capabilities of the tool.

There is also a group of studies that analyzed the further fate of patients after they had undergone a CACS examination years before. The analysis of Yamamoto et al. examined 736 patients with coronary artery disease over a period of almost seven years [7]. During the observation, 9.2% of the patients died due to cardiovascular events. The cardiovascular outcomes and all-cause mortality rates were significantly increased across the four CACS groups (0, 1–99, 100–399 and ≥400), except for the composite endpoint of cardiac death and a non-fatal myocardial infarction (MI). Tay et al. examined 934 consecutive patients after a CACS and CCTA (Coronary Computed Tomography Angiography) examination over a four-year period [13]. At least one risk factor was present in 509 of the asymptomatic participants. The patients were grouped into 0, 0–10, 10–100, 100–400 and more than 400 AU, based on their CACS. Although we used similar divisions in our research, we combined the minimal, mild and moderate groups into our G2 group. In a multivariate analysis, age, sex, hypertension, and diabetes mellitus remained significant predictors of stenosis. Age, sex, diabetes mellitus and hypertension were associated with a higher risk of significant coronary stenosis. Asymptomatic patients with a CACS of zero did not require CCTA, and therefore avoided unnecessary radiation exposure. The primary differences between the presented researches are their endpoints. Therefore, we created an analysis to document the necessity of invasive coronary artery disease treatment, while the cited authors created an analysis of the predictors of stenosis. Our research documented the significant role of a calcium score examination in predicting the necessity of invasive CAD treatment. In 25.3% of our patients, no invasive procedures (percutaneous intervention or bypass grafts) were performed during the ten-year follow-up, and only in 5.8% of the patients without calcifications in their coronaries. It is also interesting that patients with previously diagnosed diabetes had percutaneous interventions (PCI) performed, which can be associated with a faster progression of coronary artery disease. Performing percutaneous interventions earlier meant that there was not such a high intensity of multivessel atherosclerotic lesions that would qualify for CABG. The patients that were qualified for the CABG, according to the guidelines of the scientific societies, are patients with multivessel coronary artery disease. Obesity and an increased waist circumference predispose them for the occurrence of multivessel changes more often. The major limitation of the presented study is the relatively small sample size compared to similar studies such as the research of Yamamoto and McClelland [7,11]; however, our paper presents other outputs, such as an analysis that is related to the type of CAD treatment (PCI vs. CABG). It should be also stressed that the presented paper is a single center study with the limitations of this kind of study being well known. Additionally, we should also mention that in the presented paper there is lack of an unequivocal answer for the question as to whether CACS has any predictive significance above and beyond the other underlying risk factors. We will try to answer this question on a much larger population in order to adequately account for the effects of any modification or residual confounding and this paper will act as a kind of a pilot study.

Some patients had queries and fears about the use of radiation during CACS. We had to remind them that the effective dose of radiation during a calcium score examination is generally low, typically less than 1.5 mSv [14], which is the highest effective dose that is recommended by the guidelines for use in image acquisition, according to the Society of Cardiovascular Computed Tomography [15].

## 5. Conclusions

The coronary artery calcium score can potentially be an effective tool for predicting the optimal therapeutic approach for coronary artery disease in a ten-year perspective. Asymptomatic individuals with no calcifications in their coronary arteries have a very low risk of requiring an invasive treatment of coronary artery disease. Additional studies are necessary to fully support the results from this research. 

## Figures and Tables

**Table 1:** Characteristics of the patients included in the study.

Risk Factors	No Intervention	PCI	CABG	*p*
WHR	0.90 (0.864–0.920)	0.906 (0.880–0.931)	0.908 (0.872–0.942)	0.3585
Weight	74 (65–85)	85 (73.2–98.2)	96 (84.7–108.5)	0.0000
BMI	27.01 (24.39–30.04)	28.73 (26.54–32.64)	32.60 (28.19–33.90)	0.0002
Smoking YES %	22.31	29.03	15.38	0.7171
Arterial hypertension systolic (mmHg)	80 (70–85)	80 (70–90)	90 (77.5–91.2)	0.0225
Arterial hypertension diastolic (mmHg)	130 (120–140)	140 (130–150)	150 (140–160)	0.0000
Hyperlipidemia	63.84	67.74	76.92	0.6134
Diabetes	15.38	41.93	15.38	0.0037
T Family burden	71.54	64.52	55.85	0.3539
Physical activity	32.31	45.16	46.15	0.2892
Waist	89.50 (82–100)	95 (92–102)	102 (94.50–109)	0.0021
Age	58 (54–63)	61 (56–69)	57 (54.25–62.75)	0.0890
CACS	9.5 (0–172)	598 (97–918)	577 (421.75–975)	0.0000
Place of living (city)	85.38	77.42	92.31	0.3930

WHR = Waist–Hip Ratio, CACS = Coronary Artery Calcium Score, PCI = Percutaneous Coronary Intervention, CABG = Coronary Artery Bypass Graft.
